# A Theoretical Model: Elastic Analysis of the Evolution of the Crypt Opening Between the Fundic Gland and the Pyloric Gland

**DOI:** 10.3389/fphys.2018.01388

**Published:** 2018-10-02

**Authors:** Fei Xiong, Xiao Gang Liu

**Affiliations:** Department of Gastroenterology, Sichuan Academy of Medical Sciences & Sichuan Provincial People's Hospital, Chengdu, China

**Keywords:** crypt opening, fundic gland, multiple white flat lesions, the Euler force, epithelial-mesenchymal transition

## Abstract

In recent years, with the development of magnified endoscopic technology, the microstructure of the gastric mucosa surface has been widely studied. However, it is unclear why the crypt opening shape of the fundic gland is different from that of the pyloric gland. We attempted to explain the problem by means of physical concepts, mathematical tools and some pathological perspectives. We first constructed an “L” type tubular structure on the basis of the pathology of the gastric mucosa and some geometric principles and then analyzed the distortion of marginal crypt epithelia after we added cells in the model via the mechanism of continuous regeneration. Finally, we determined that the crypt opening shape of the pyloric gland is derived mathematically from that of the fundic gland with the help of the idea of the Riemann sum. According to the derivation of the Euler force, it is possible that the epithelial-mesenchymal transition (EMT) protects the integrity of the gastric mucosa. Our model suggests that the evolution of the fundic gland and the pyloric gland triggers the EMT via elastic deformation. The basic logic of our model is the principle of least action.

## Introduction

Magnified endoscopic technological (Yao, 2014) development in recent years has made observing the microstructure of the gastric mucosa easier than with a scanning electron microscope. Based on extensive clinical studies (Yao, 2014, 2015), structural changes within the gastric mucosal surface are accepted universally as an important reflection of its physiological function and pathological effects in the stomach. As the site through which gastric acid and digestive enzymes exit (Johansson et al., 2000; Phillipson, 2004), the gastric crypt opening plays a vital part in the micro-surface of the stomach. Understanding this process on a microstructural level will help us better understand metaplastic (chronic) atrophic gastritis (Jense and Feldman, 2016) and early gastric cancer (Shimizu et al., 1995).

**Figure 1:**
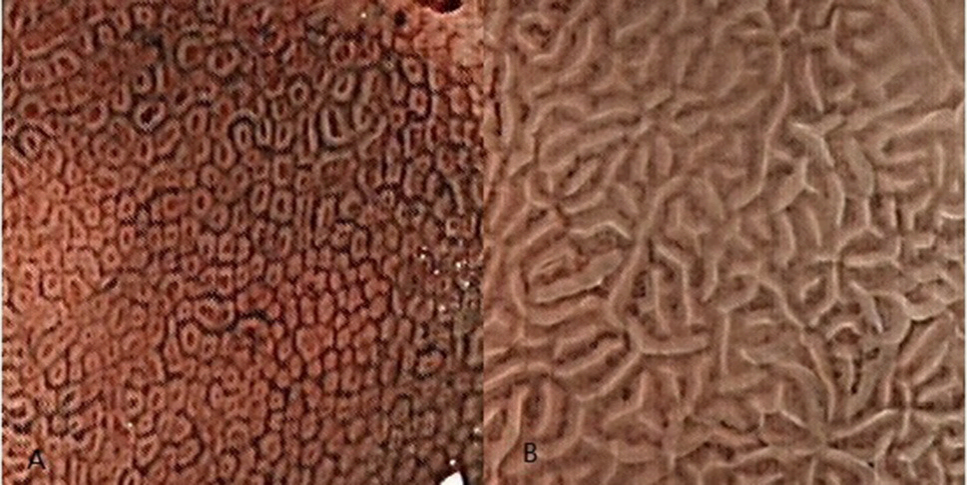
The Crypt opening morphology of the gastric mucosa. These images were taken using magnified endoscopic technology with narrow-band imaging (NBI-ME). **(A)** The subepithelial capillary network (SECN) looks like a honeycomb in the gastric body, and we can see many approximate round or oval dots in the middle of the honeycombs. These black dots are the crypt openings of fundic glands. **(B)** Although we cannot see the crypt opening in the gastric antrum mucosa, gastric crypt openings are approximately linear or have a reticular groove based on NBI-ME and pathological examination. These openings are hidden between marginal crypt epithelia (white stripes).

The traditional view of pathology says that the crypt opening in the stomach should be approximately round or oval. The crypt opening morphology of the fundic gland certainly matches this view based on the magnified endoscopy (ME) photographic data (Yao, 2014; Boeriu et al., 2015; Muto et al., 2015) and scanning electron microscopy (SEM) (Sugimoto and Ogata, 1989), but the gastric crypt opening shows a completely different shape in the gastric antrum (Yao and Oishi, 2001; Niwa et al., 2008; Yao, 2014). If we observe the antral gastric mucosa using ME with narrow-bandimaging (NBI-ME) (Figure 1B), the crypt openings are difficult to see because the rugged intervening section between them that is located side-by-side can cause marginal crypt epithelia (MCE) to cover the crypt openings. Some researchers (Yao, 2014, 2015; Muto et al., 2015) have found that using a combination of two kinds of technology, magnified examination with some assistive technology, and examining the cross-section via microscope can help locate the antral gastric crypt openings. As technology advances, a growing number of studies (Yao, 2014, 2015; Boeriu et al., 2015; Muto et al., 2015) have suggested that gastric crypt openings are approximately linear or have a reticular groove. In addition, the discovery of the light blue crest (Uedo et al., 2006) using NBI-ME indirectly supports this idea.

From the above studies, we can conclude that a linear or reticular groove is the morphological feature of crypt openings in the gastric antrum mucosa. Although studies of human gastric mucosa have evolved significantly in just the past decade, some key questions are still difficult to answer. Why is the gastric crypt opening approximately linear or a reticular groove in the gastric antrum mucosa? Why are the micro-surfaces of the multiple white and flat elevated lesions very similar in the gastric antrum mucosa and gastric fundic mucosa? It is possible that ME or pathology cannot answer these questions. Thus, these problems may need to be answered using different methods, such as a constructed elastic mechanics and mathematical modeling.

### Model

From a physiological and histological (Rotterdam and Enterline, 1989) perspective, the tubular structure is an important structure in the gastric mucosa. Among the spectrum of gastric diseases, intestinal gastric cancer (IGC) requires a “cascade” of events, and chronic active non-atrophic gastritis can progress over time to invasive carcinoma (Correa, 1992) (Figure S1). IGC has a glandular structure and replaces the full mucosa layer of the surrounding glands (Oyama, 2016). The evolution of IGC implies that the tubular structure is the core structure in the gastric mucosa. Furthermore, there is a high rate of IGC in specific high-risk areas (Crew and Neugut, 2006). Therefore, as a first step (Brannan and Boyce, 2015), we chose the tubular structure to construct our elastic mechanics and mathematical model. Because the gastric body crypt openings are round or oval, we used the fundic gland as our model's starting point. The gastric acid secretions from several glands reach the gastric lumen through only one pit (McDonald et al., 2008; Hoffmann, 2015). We call this system a gastric unit. However, we need to moderate abstract scientific methods to grasp the essence of our main question (i.e., the mechanism of forming the crypt opening of the gastric mucosa) and to ignore many other less important factors in our model, such as the complex, 3D structure of the gastric unit. Therefore, for simplification, let us suppose that a single pit connects a single gland.

### “L” type tubular structure

Foveolar cells, chief cells, parietal cells, and other cells comprise the gastric gland. These simple columnar epithelial cells share a common feature: nuclear polarity. The major axis of a normal cell nucleus is always perpendicular to the basement membrane. Considering this fact from the perspective of a 2D plane, a closed curve is a reasonable structure when a liquid needs to be transported and stored in a biological system. Many cross-sectional diagrams of pathological digestive tracts also confirm this view. Therefore, the first step in our model is to find the most suitable geometric shapes of these closed curves. As a complex biological system, the real geometric shapes of these closed curves are influenced by many factors. Differing stiffness of the extracellular matrix can induce morphological differentiation of stem cells (Engler et al., 2006). Epithelial cells feature a distinguishing characteristic, epithelial-mesenchymal transition (EMT) (Kalluri and Weinberg, 2009), and the extracellular matrix (ECM) and glandular cell's mechanical force [produced by E-cadherin (Guilford et al., 1999) and called an ideal state] are undoubtedly the major contributors to the geometric shapes of these closed curves. Thus, the biological system can be written as Equation (1):

(1) $$\begin{array}{r} \frac{dG_{real}}{dt}=\frac{dG_{ideal}}{dt}+\frac{\partial E}{\partial T}\cdot \frac{dG_{ideal}}{dE} \end{array}$$

*G*_*real*_ is the real geometric shape of these closed curves. *G*_*ideal*_ is the ideal geometric shape of these closed curves, and it is influenced by the ECM and time. The value of its function is equal to *dG*_*ideal*_, where *t* is time (Figure S1). EMT suggests that *G*_*real*_ plays a major role in the early evolution of our model. Let us suppose that *t* approaches 0; then, we obtain Equation (2):

(2) $$\begin{array}{r} \frac{dG_{real}}{dt}\approx \frac{dG_{ideal}}{dt} \end{array}$$

**Figure 2:**
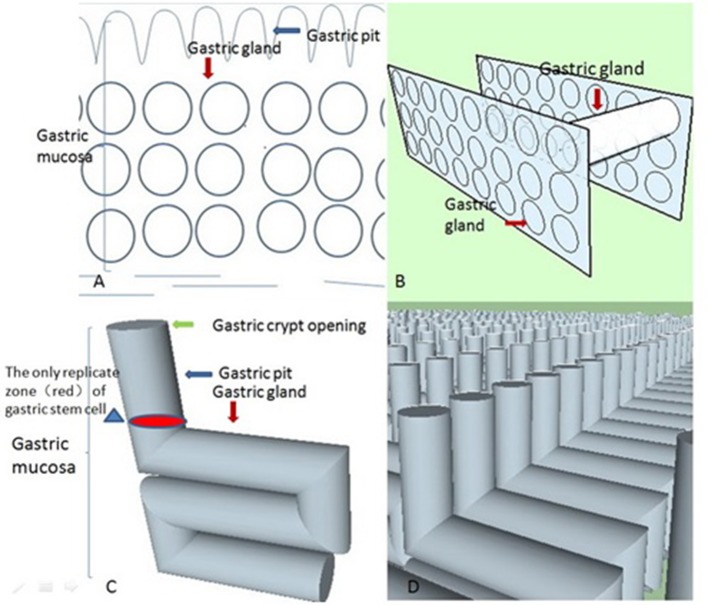
These four conceptual diagrams show the derivation process of these stereoscopic structures of gastric glands in an ideal state based on the principle of least action. **(A)** According to isoperimetric inequality, we delineated an ideal geometrical morphology of the gastric gland in 2D space. **(B)** Let us suppose there are many similar 2D spaces (like Figure 2A) overlapping along a straight line that is parallel to the gastric mucosal surface, which gives us many cylinders. From the least action principle, these cylinders are parallel to the gastric mucosal surface. **(C)** When we unite the geometric shapes of the gastric body's gastric pits with these cylinders, we can obtain an “L” type tubular structure of the gastric gland in the ideal state. **(D)** The “L” type tubular structure of the gastric gland fills the whole mucosa in the ideal state. Its real state is disorganized and chaotic. The neat rows of these “L” type tubular structures are only easy to observe (the distribution of these structures does not contribute to the final result). A gastric unit consists of several glands that open into a common pit. However, if we apply that definition, our model becomes more complex in what was already a very complex environment. Our aim is to find the mechanism for the formation of the crypt opening of the gastric mucosa, not the stereoscopic structures of the gastric gland. For simplification, we assume that a single pit is connected to a single gland.

The biological system should be approximately in an ideal state in the initial stage based on the principle of approximation in calculus (Thomas et al., 2004). For a greater understanding of our model, we first need to construct it in an ideal setting. Prior to finding the most suitable geometric shapes of these closed curves, we need to use the principle of least action (Feynman et al., 1970; Xiong, 2015) as the basis for our model because the principle is so universal in nature. That is, we need to show that the tubular structure conforms to the least action principle by modeling crypt opening morphogenesis. The gastric unit needs to deliver gastric acid and pepsin to the gastric mucosa. The 2D plane (i.e., the cross-section) represents the instantaneous state of acid and pepsin secretion compared with the 3D space (Xiong, 2015). Therefore, these closed curves represent an instantaneous state of transportation of the liquid. The least action principle examines these closed curves that sense all geometric shapes and chooses the one with the least action. From a transport efficiency perspective, the larger the closed curve, the higher the gastric gland's transport efficiency. According to isoperimetric inequality, the assumptions for the circumference of the closed curve are fixed in our model, and the most suitable geometric shape of these closed curves is a circle. In short, the circle has the least action in a 2D plane from a transport efficiency perspective. Therefore, we can show the geometric pattern of the cross-sectional diagram in an ideal state (Figure 2A). Now, suppose that we imagine that many similar 2D spaces overlap along a straight line parallel to the gastric mucosal surface. Thus, we can gain many cylinders (Figure 2B). However, from our pathologist's perspective (Montgomery and Voltaggio, 2011), these gastric glands are a coiled and complex structure rather than a simple structure consisting of straight tubes that are perpendicular to the gastric mucosa in 3D space. Therefore, according to the principle of least action, we need to identify what these cylinders' spatial arrangement is in an ideal setting. From an evolutionary perspective, gastric acid secretion may put humans at an evolutionary advantage. We need to produce enough gastric acid to filter microbes in a short time (Beasley et al., 2015).

**Figure 3:**
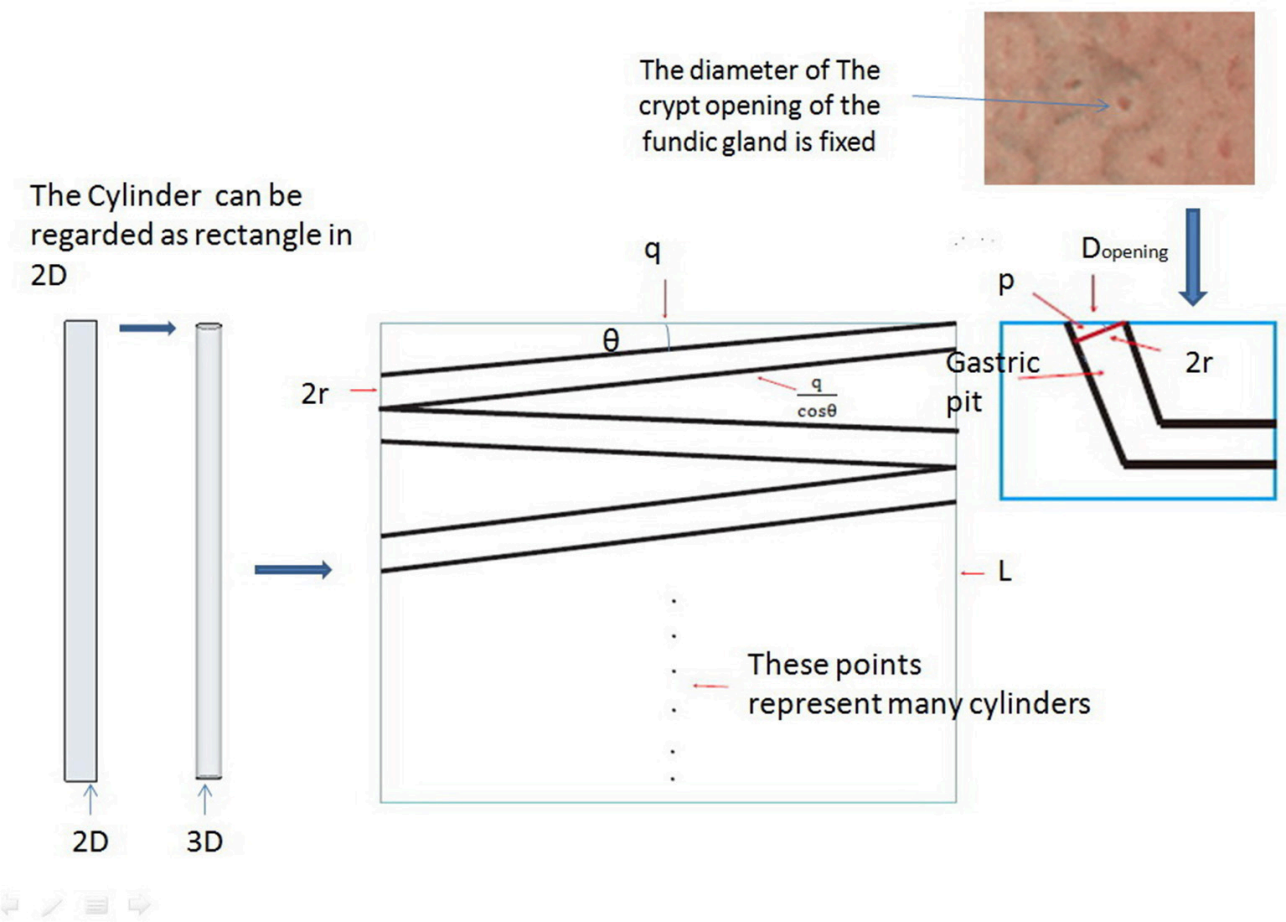
We can think of cylinders as rectangles in a 2D plane. The middle part of the picture shows the rectangle plane distribution at any angle θ_*k*_ (*k* = 1, 2, 3 … n), and we can demonstrate that these rectangles are parallel to q based on the principle of least action with the help of some properties of triangles and the definition of area.

We can prove that these cylinders are parallel to the gastric mucosa with the help of some properties of triangles and the fact that the pit tends to be perpendicular to the x-axis with the help of the least action principle (extended mathematical analysis procedure and Figure 3). Therefore, we can produce a model of an “L” type tubular structure (Figure 2).

### Multiple flat white lesions

**Figure 4:**
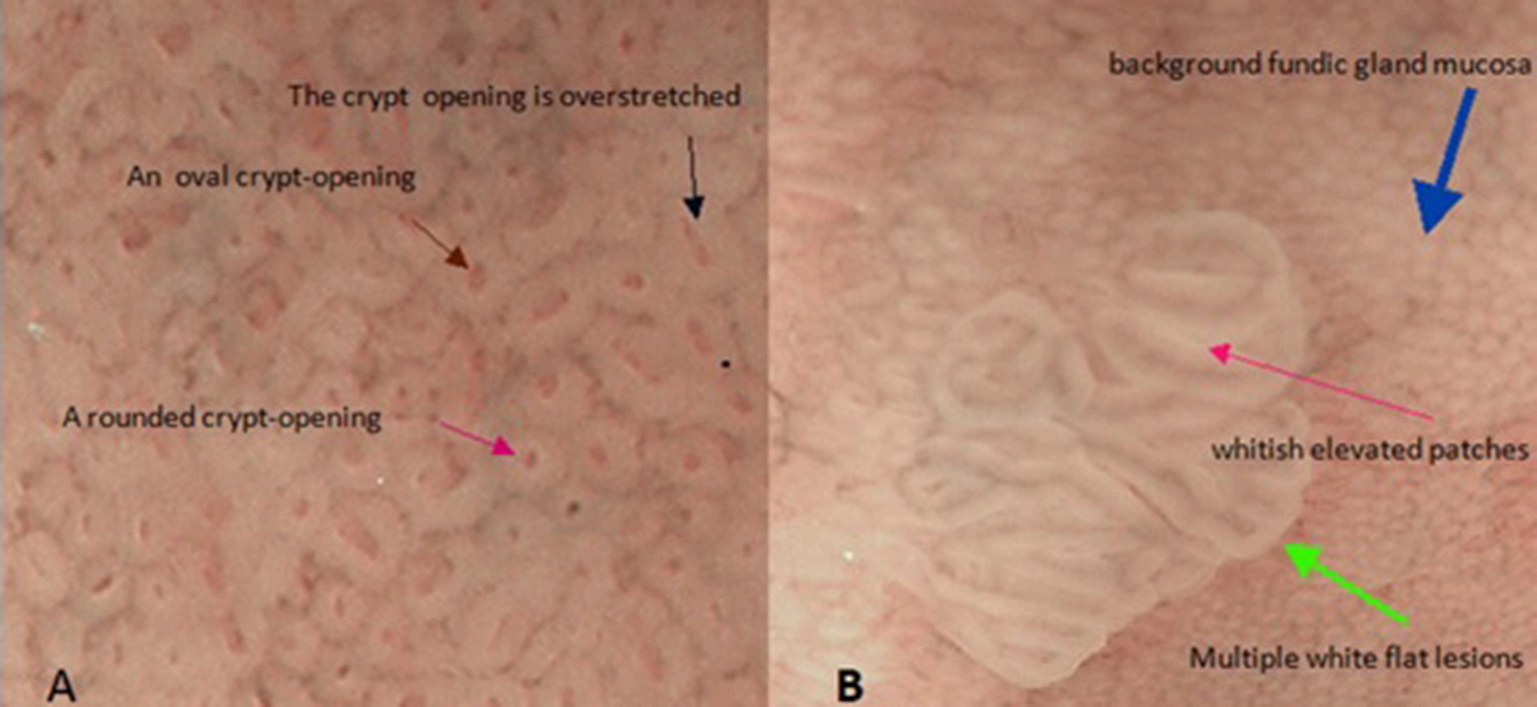
These two pictures show the mucosal surface structure of the fundus of the stomach with the help of magnified endoscopy with narrow-band imaging (NBI-ME). **(A)** There are some different morphological characteristics of gastric crypt openings. **(B)** This image shows the morphological characteristics of a flat white lesion. There are huge differences in the epithelial structures between background fundic gland mucosa and multiple white flat lesions.

We now return to our original question. Thus far, our model has been based on the fundic gland's microstructure. Therefore, the next logical thing to do is to determine the structure of the mucosa surface as the connection between fundic glands and pyloric glands from a pathological perspective. Fortunately, multiple white flat lesions (Uedo et al., 2017) in the gastric corpus may be able to play this role (Figures 1B, 4). If we look closely at multiple white flat lesions, it is safe to assume that the micro-surface structures of the multiple white flat lesions are similar to the antral gastric mucosa. Furthermore, if this assumption is true, then the morphological features of the crypt opening on the multiple white flat lesions are linear or a reticular groove, just as with the antral gastric mucosa.

In addition, as described by Uedo et al. (2017), multiple white flat lesions are hyperplastic lesions. The characteristics of hyperplastic lesions suggest that continuous regeneration (Hoffmann, 2008) via proliferation and differentiation of stem cells is responsible for multiple white flat lesions. Additionally, dysregulated regeneration causes intestinal metaplasia. The appearance of whitish elevated patches in the antrum often represents intestinal metaplasia on magnified narrow-band imaging. The same characteristic is observed on multiple white flat lesions (Uedo et al., 2017). This coincidence allows us to associate continuous regeneration with multiple white flat lesions. Hence, in our model, we need to demonstrate only that the morphological features of the crypt opening on the multiple white flat lesions are linear or a reticular groove based on continuous regeneration.

## Results

### The motion of the epithelium in the gastric fundus and body

**Figure 5:**
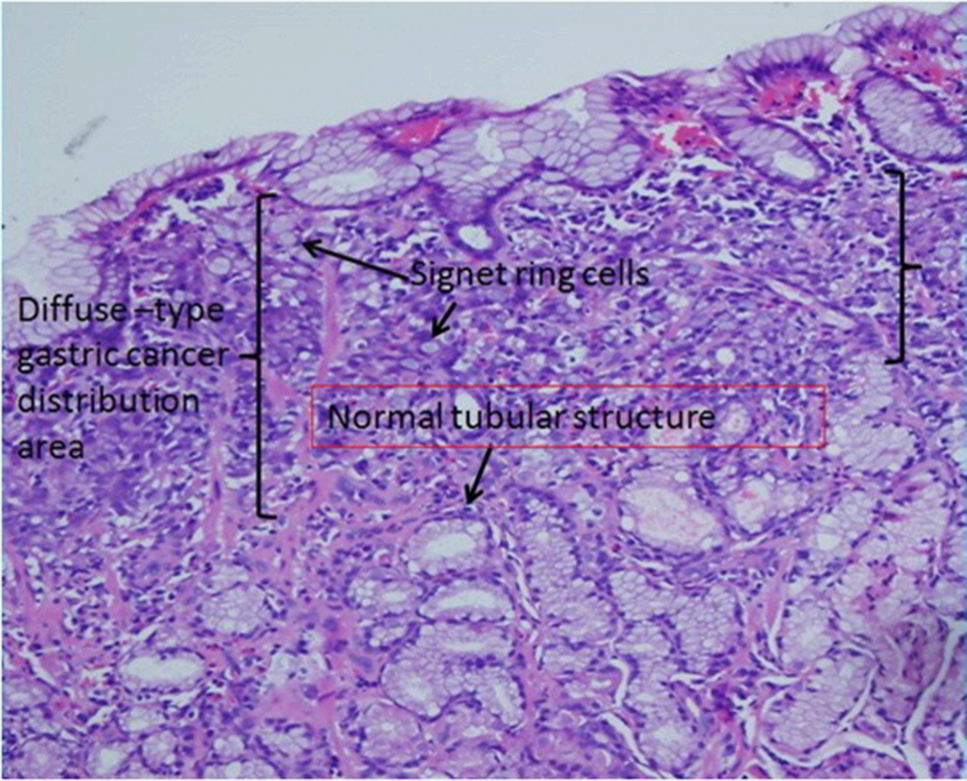
Gastric signet ring cell carcinoma confined to the mucosal layer in the picture. If we carefully observe the diffuse-type gastric cancer distribution area, the lateral length of the distribution area is far longer than the longitudinal length. This characteristic suggests that diffuse-type gastric cancer spreads laterally in the pathological section. In addition, based on our model, we find many normal tubular structures below the diffuse-type gastric cancer distribution area.

Approximately 60 years ago, Leblond et al found that the isthmus and neck are the major sites of gastric stem and progenitor cells (Stevens and Leblond, 1953). However, it is difficult to determine the isthmus and neck's location because the gastric gland is a complex spatial structure. Fortunately, for our model, we can look for some clues about the isthmus and neck's location based on pathology. According to a Japanese experts' view (Oyama, 2016), each cancer cell in poorly differentiated adenocarcinoma spreads laterally at the isthmus and neck of the gland. As shown in the “L” type tubular structure, let us suppose that gastric stem cells are distributed into rings and that only two geometric types of replication zones of gastric stem cells' spatial position are possible: a closed ring in the horizontal orientation and a closed ring in the vertical orientation. If the latter is true, half of the cancer cells move more vertically in pathological sections. Therefore, the latter is far removed from the direction of the poorly differentiated adenocarcinoma's invasion in the cross-sectional diagram (Figure 5). In addition, we already know that several glands share a single pit. According to gland fission's formation mechanism (McDonald et al., 2008), if the replication zone of gastric stem cells' spatial position is in the horizontal orientation, a new gland's development will be blocked by the dense structure of the gastric unit. Thus, it is reasonable that we place the replication zone of gastric stem cells at the bottom of the pit in our model (Figure 2C).

**Figure 6:**
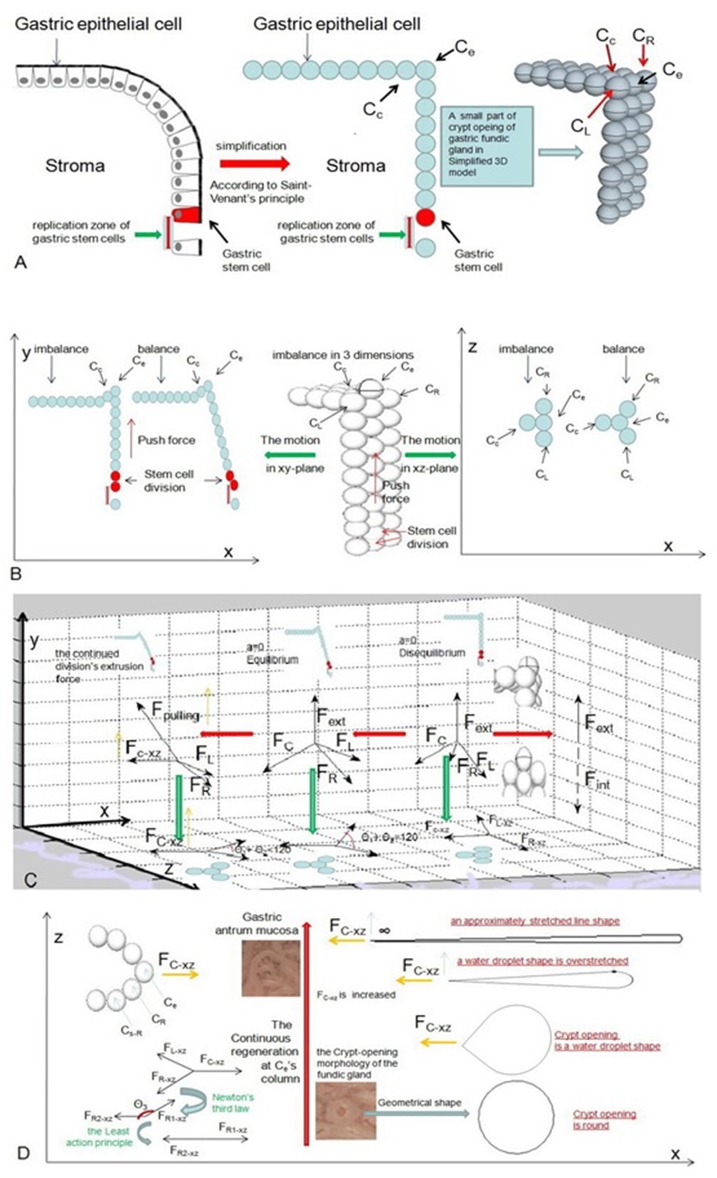
These pictures show the motion of the epithelial cell in an ideal state. **(A)** We need to simplify these cells into roundness based on Saint-Venant's principle and suppose that cells are arranged in rows and columns. **(B)** When a gastric stem cell splits and forms two new gastric epithelial cells within *C*_*e*_'s column, these forces act across*C*_*e*_ and are analyzed at the initial stage. **(C)** Our model shows the changes in these forces and the changes in the position of *C*_*e*_ for balance. **(D)** The crypt opening demonstrates an approximately stretched line shape when *F*_*C*−*xz*_ is infinite.

Simple columnar epithelial cells belong to every part of the stomach. For our model, in an ideal state, the only mechanical force holding simple columnar epithelial cells together is E-cadherin (Guilford et al., 1999). To better understand the mechanical force that acts on the gastric epithelium, it is important to simplify these cells into roundness based on Saint-Venant's principle and to suppose that cells are arranged in rows and columns (Figure 6A). According to Natalia Guz et al.'s research (Guz et al., 2014), our model can be viewed as an elastic body. We need to know that the elastic body also satisfies the least action principle (Feynman et al., 1970). To minimize energy, the elastic body's internal forces must be in equilibrium. Now suppose we have an epithelial cell on the MCE of a fundic gland. For our model, it is located at the top edge of the vertical cylinder. We called it *C*_*e*_, and if *C*_*e*_ is in equilibrium, we have:

(3) $$\begin{array}{r} F_{int}=0 \end{array}$$

where *F*_*int*_ is the total internal force acting on *C*_*e*_. Suppose gastric stem cells are distributed in a ring at the bottom of a pit in our model. For our model, there is a one-to-one correspondence between each gastric stem cell and each column of epithelial cells in the vertical cylinder. Let us denote the two cells on the right and left side of *C*_*e*_ by *C*_*R*_ and *C*_*L*_ at MCE sites (Figure 6A). At least one epithelial cell is also connected to *C*_*e*_ at the gastric epithelium besides the MCE. We denote it by *C*_*c*_ for our model, and we can use the regeneration mechanism of the mucous epithelia, i.e., continuous regeneration via the differentiation of stem cells (Hoffmann, 2008), in the following way: we consider that a gastric stem cell splits and forms two new gastric epithelial cells only at *C*_*e*_'s column (Figure 6B). For *C*_*e*_'s column, the added cell can generate external force, which acts from a distance on *C*_*e*_ to produce a force per unit volume *f*_*ext*_. Owing to elastic strain energy attenuating with distance (Toupin, 1965), let us suppose that *C*_*e*_ will deform more or less under the action of the external force, *F*_*ext*_. Then, we can write *F*_*ext*_ as

(4) $$\begin{array}{r} F_{ext}{=\int f}_{ext}dV \end{array}$$

where *V* is these cells' average volume.

The elastic body must adjust itself to an equilibrium because of the least action principle. Therefore, the internal stresses on it must also adjust themselves to minimize energy (Feynman et al., 1970). Furthermore, *F*_*ext*_ should counteract the force *F*_*int*_ from the neighboring cells, which act across *C*_*e*_. Let us suppose that these cells are not in equilibrium. If they are moving, we have

(5) $$\begin{array}{r} {\;F}_{int}+F_{ext}=\int \rho \cdot a\;dV \end{array}$$

where ρ is the density of these cells, and *a* is their acceleration. We can now combine Equations (4) and (5) to yield

(6) $$\begin{array}{r} F_{int}=\int \left(f_{ext}+\rho \cdot a\right)dV \end{array}$$

When these cells are in equilibrium, there is no acceleration. Then, Equation (5) is written as

(7) $$\begin{array}{r} \text{a}=0\Rightarrow F_{int}+F_{ext}=0 \end{array}$$

In other words, we can change Equation (7) to

(8) $$\begin{array}{r} F_{int}{=\int f}_{ext}dV \end{array}$$

These equations tell us how the external force from cell division is related to the sum force *F*_*int*_ from the neighboring cells, which act across *C*_*e*_. As shown in an earlier chapter, the cell adhesion force (Guilford et al., 1999) is only an internal force of the elastic body in an ideal state. According to the definition of vectors, both *F*_*int*_ and *F*_*ext*_ are represented by a directed line segment. Equation (8) suggests that the vector lengths are equal. Now let us put our model into rectangular coordinates. The x-axis is parallel to the mucosal surface, the y-axis is perpendicular to the mucosal surface and the z-axis is perpendicular to the xy-plane:

(9) $$\begin{array}{r} F_{int-x}{=\int f}_{ext-x}ds \end{array}$$

(10) $$\begin{array}{r} F_{int-y}{=\int f}_{ext-y}ds \end{array}$$

(11) $$\begin{array}{r} F_{int-z}{=\int f}_{ext-z}ds \end{array}$$

where *s* is a curve. Equation (9) through Equation (11) suggest the elastic body is in equilibrium in the x, y, and z directions. There are 3 directions that we can examine to help us understand how *C*_*e*_ reaches equilibrium after a gastric stem cell finishes a division at *C*_*e*_‘s column.

### The Y-axis

When a gastric stem cell splits and forms two new gastric epithelial cells, only *C*_*e*_ is produced by *C*_*e*_'s column. The y-axis force toward *C*_*e*_ is produced by an extrusion force from *C*_*e*_‘s column (Figure 6C). The force produces the initial displacement of *C*_*e*_ on the y-axis. The elastic body is not in equilibrium in the initial stage. According to Equations (7) and (8), we have

(12) $$\begin{array}{r} a_{y}=0\Rightarrow F_{ext}=F_{R-y}\cos\theta_{R-y}+F_{L-y}\cos\theta_{R-y}+F_{C-y}\cos\theta_{R-y} \end{array}$$

where *a*_*y*_ is a component of acceleration on the y-axis. After *C*_*e*_ is in equilibrium, *F*_*R*_, *F*_*L*_, and *F*_*C*_ indicatethat some pulls from *C*_*c*_, *C*_*R*_, and *C*_*L*_ act on *C*_*e*_. θ_*R*−*y*_, θ_*L*−*y*_, and θ_*C*−*y*_ represent the angles between these pulls among *C*_*c*_, *C*_*R*_, *C*_*L*_ and the y-axis.

### The Z-axis

Because *C*_*R*_ and *C*_*L*_ are located on either side of *C*_*e*_, it is reasonable that their components are equal on the z-axis. We will not go into detail in our model because they donot impact the distortion of the elastic body.

### The X-axis

Let us suppose that *C*_*R*_, *C*_*L*_, and *C*_*e*_ lie on one line based on the approximation principle. Only one component can generate a pulling force from *C*_*c*_ on the x-axis. We obtain new displacement of *C*_*e*_ on the x-axis. As mentioned previously, the energy of the elastic body must be minimized. Now, we project *F*_*R*_ and *F*_*L*_ and *F*_*C*_ onto the xz-plane, and we can obtain three components of *F*_*R*_, *F*_*L*_, and *F*_*C*_ in the xz-plane. For our model, *F*_*R*−*xz*_, *F*_*L*−*xz*_, and *F*_*C*−*xz*_ are adapted to represent each of these components. To obtain equilibrium, *F*_*R*−*xz*_ and *F*_*L*−*xz*_ gradually move inward as *F*_*C*−*xz*_ increases until the sum component force of *F*_*R*−*xz*_ and *F*_*L*−*xz*_ on the x-axis is equal to *F*_*C*−*xz*_. Therefore, we have

(13) $$\begin{array}{r} F_{C-xz}=F_{R-xz}\cos\theta_{1}+F_{L-xz}\cos\theta_{2} \end{array}$$

where θ_1_ *and θ*_2_ are the angles between *F*_*R*−*xz*_ and *F*_*L*−*xz*_ and the x-axis, respectively.

Suppose that we imagine that the gastric stem cell continues cell division. The number of epithelial cells continues to increase in *C*_*e*_'s column. The continued division can result in an increase in *F*_*C*−*xz*_. Finally, we have (Figure 6C)

(14) $$\begin{array}{r} {\;\theta }_{1}+\theta_{2}=120^{{}^{\circ}}\Rightarrow F_{C-xz}=F_{R-xz}=F_{L-xz} \end{array}$$

The equation represents these forces' equilibrium state in the xy-plane. However, if cell divisions continue to occur, they could cause an incline at *C*_*e*_'s column due to the displacement of *C*_*e*_. Therefore, θ_1_+θ_2_ reach 0, and *F*_*C*−*xz*_ will continue to increase due to a component of the continued division's extrusion force in the direction of the x-axis (Figure 6C). We call this force *F*_*pulling*_. As a result, based on the least action principle, the elastic system has to further decrease the value of θ_1_+θ_2_ because the sum component force of *F*_*R*−*xz*_ and *F*_*L*−*xz*_ on the x-axis counteracts *F*_*C*−*xz*_ with minimal effort. Now we use *C*_*s*−*R*_ to represent the first cell behind *C*_*R*_ on MCE. According to Newton's third law, we indicate that the counterforce of *F*_*R*−*xz*_ acting on *C*_*R*_ as *F*_*R*1−*xz*_ in the xz-plane. A force from the junction between *F*_*R*1−*xz*_ and *F*_*R*2−*xz*_ as θ_3_ also exists. Finally, we ignored the influence of other junctions between *C*_*R*_ and other cells. When our system is in equilibrium, we have

(15) $$\begin{array}{r} F_{R1-xz}=F_{R2-xz}\cos\left(\pi -\theta_{3}\right) \end{array}$$

Let us assume that the value of *F*_*C*−*xz*_ tends to be infinite and that cellular junctions among epithelial cells donot break. According to the least action principle, we can write

(16) $$\begin{array}{r} F_{C-xz}↑\Rightarrow F_{R-xz}↑\Rightarrow \theta_{3}\approx \pi \Rightarrow F_{R1-xz}=F_{R2-xz} \end{array}$$

Therefore, the crypt opening of the fundic gland has a water droplet shape in the xz-plane (Figure 6D). θ_1_+θ_2_ approaches zero, and the crypt opening demonstrates an approximately infinitely stretched line shape under magnifying endoscopy (Figure 6D).

### Buckling of the gastric epithelium

**Figure 7:**
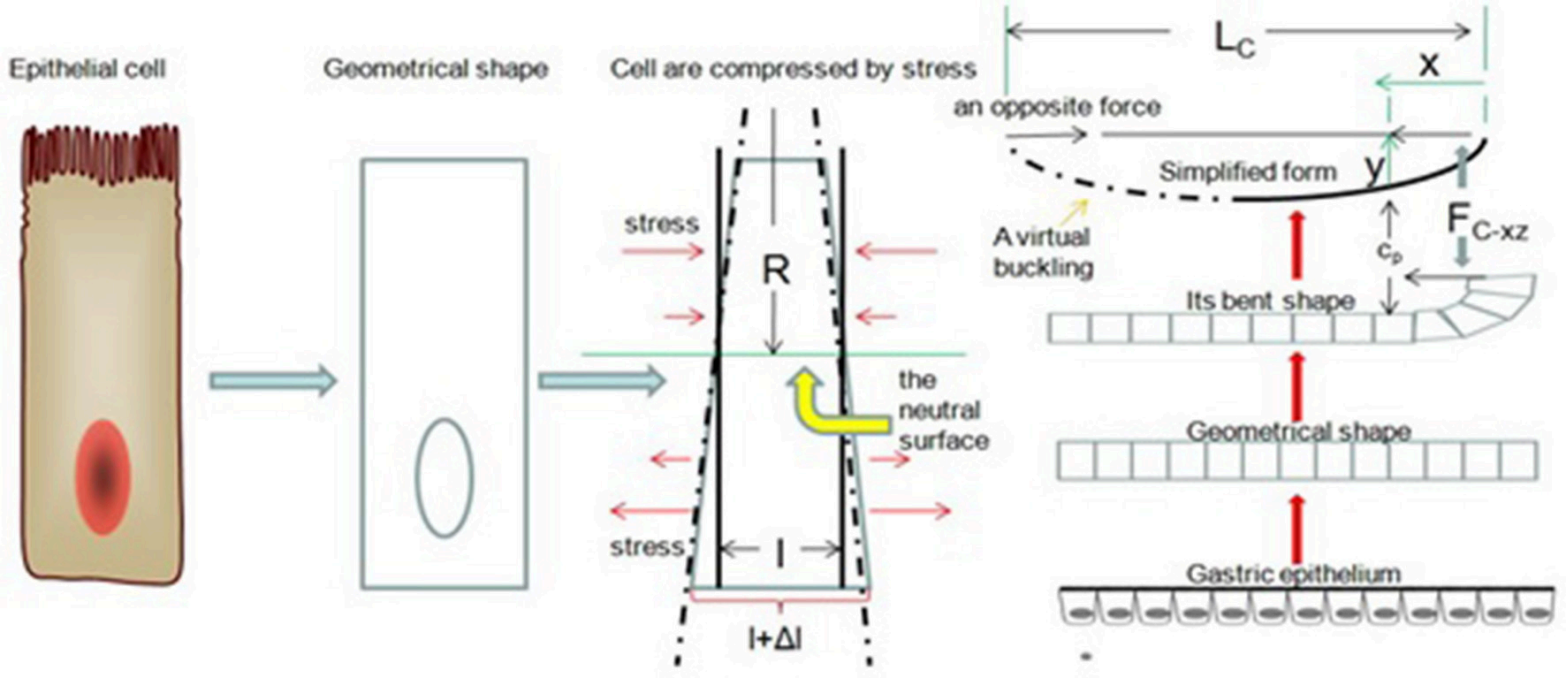
Buckling of the gastric epithelium. The cell components above the neutral surface are compressed, and the cell components below the neutral surface are stretched. When *F*_*C*−*xz*_ is increasing, the gastric epithelium can be simplified as a bending line.

In the previous model, we ignored a problem: the gastric epithelium will break when *F*_*ext*_ reaches a certain value. Next, we will address this problem from a classical theory of elastic mechanics, Hooke's law. Now, we observe our model from the xy-plane. Suppose we take a segment of gastric epithelial cells of length l. These cells are again columnar. When cell division occurs within *C*_*e*_'s column, we expect a small bending of the gastric epithelium. There exists a surface of simple columnar epithelial cells that is parallel to the x-axis, and its length is *l*. Thus, cell components above the surface are compressed, and the cell components below it are stretched. We called the surface a neutral surface (Feynman et al., 1970). Now we set Δ*l* as the longitudinal stretch, *y* as the height between the neutral surface and normal gastric mucosa, and *R* as the radius of curvature of the gastric epithelium. Based on geometry, we have (Figure 7)

(17) $$\begin{array}{r} \frac{\Delta l}{l}=\frac{y}{R} \end{array}$$

A unit area in a columnar cell *F*_*cell*_ is proportional; therefore, according to Hooke's law (Feynman et al., 1970), the force per longitudinal section (xz-plane)

(18) $$\begin{array}{r} \frac{\Delta F_{cell}}{\Delta A_{cell}}=E\cdot \frac{\Delta l}{l}=E\cdot \frac{y}{R} \end{array}$$

where *E* is Young's modulus, and Δ*A*_*cell*_ is a longitudinal section (xz-plane) of unit area of a columnar cell, which is the area between a columnar cell's free surface and the neutral surface. For a small bend, the neutral line is near the middle of the cross-section (xz-plane), and *y* can be approximated as the distance between the neutral surface and an epithelial cell's basal surface. Therefore, we can compute the total bending moment:

(19) $$\begin{array}{r} \tau =\int_{longitudinal\;section\left(xz-plane\right)}ydF_{cell} \end{array}$$

where τ is the bending moment. From Equation (18), we have

(20) $$\begin{array}{r} \tau =\frac{E}{R}\int_{longitudinal\;section\left(xz-plane\right)}y^{2}dA_{cell} \end{array}$$

According to the definition of moment of inertia (Feynman et al., 1970), we have

(21) $$\begin{array}{r} I=\int y^{2}dA_{cell} \end{array}$$

where *l* is the moment of inertia. Its principal axis is parallel to the x-axis and through the cell's barycenter. Then, the bending moment is

(22) $$\begin{array}{r} \tau =\frac{EI}{R} \end{array}$$

Suppose that we imagine that an opposite force (equal to *F*_*C*−*xz*_) is at the other end of the segment of the gastric epithelium in the xy-plane (Figure 7). We call any one cell of the gastric epithelial cells *C*_*p*_. Therefore, the bending moment τ of *C*_*p*_ is

(23) $$\begin{array}{r} \tau_{p}=F_{C-xz}\cdot y_{p} \end{array}$$

where *y*_*p*_ is the moment arm, the perpendicular distance between *C*_*p*_ and a line along the direction of *F*_*C*−*xz*_. Using equation (20), we rewrite the equation as

(24) $$\begin{array}{r} \frac{EI}{R}=F_{C-xz}\cdot y_{p} \end{array}$$

When our model adds a new epithelial cell at *C*_*e*_' s column, we can see the deformation as a small bend. According to the definition of curvature (Thomas et al., 2004), we can take

(25) $$\begin{array}{r} \frac{1}{R}=\frac{dT_{curvature}}{dS_{curvature}}=\frac{d^{2y_{p}}}{d{x_{p}}^{2}} \end{array}$$

where *T*_*curvature*_ is a unit tangent's vector of the gastric epithelium, and *S*_*curvature*_ *S*_*curvature*_ is the arc length parameter. Then, we have

(26) $$\begin{array}{r} \frac{d^{2y_{p}}}{d{x_{p}}^{2}}=\frac{F_{C-xz}\cdot y_{p}}{EI} \end{array}$$

We can see the right side of the equation is equal to an inverted sine wave equation. Now, we can set the connecting line between the ends to *L*_*c*_. For convenience, let us assume that the wavelength is equal to *L*_*c*_ and that *L*_*c*_ is twice as long as *l*

(27) $$\begin{array}{r} y_{p}=K\sin\frac{\pi x_{p}}{L_{c}} \end{array}$$

where *K* is constant. Taking the derivative, we have

(28) $$\begin{array}{r} \frac{dy_{p}}{dx_{p}}=-K\frac{\pi }{L}\cos\frac{\pi x_{p}}{L_{c}} \end{array}$$

Taking the derivative again, we get

(29) $$\begin{array}{r} \frac{d^{2}y_{p}}{{dx_{p}}^{2}}=\;K\frac{\pi^{2}}{{L_{c}}^{2}}\sin\frac{\pi x_{p}}{L_{c}} \end{array}$$

Using Equation (25):

(30) $$\begin{array}{r} \frac{d^{2}y_{p}}{{dx_{p}}^{2}}=\frac{\pi^{2}}{{L_{c}}^{2}}\cdot y_{p} \end{array}$$

We put this equation into Equation (25), and we can see

(31) $$\begin{array}{r} F_{C-xz}=\frac{\pi^{2}}{{L_{c}}^{2}}\cdot EI \end{array}$$

The force of the equation is more commonly known as the Euler force (Feynman et al., 1970). The equation shows the force is independent of *y*_*p*_ for small bending. In other words, if the force is less than the Euler force, it is impossible for the gastric epithelium to break. It is logical to keep *F*_*C*−*xz*_ in the Euler force's vicinity with the help of some mechanisms so that our gastric epithelium does not break during continuous regeneration. Now let us return to the multiple white flat lesions. Based on the research of Uedo et al. (2017), there are two biological structures in its pathological sections: tubular structures and mesenchyme. The former structures are produced by continuous regeneration; we need to understand the source of multiple white flat lesions' mesenchyme. According to renal fibrosis research, renal fibrosis is induced by pressure force (Hoffman et al., 2006; Broadbelt et al., 2007; Chen et al., 2014). For our model, logic suggests that the pressure force is equal to the stress of the deformation of the gastric epithelium. In short, if $F_{C-xz}>\frac{\pi^{2}}{{L_{c}}^{2}}\cdot EI$, EMT will be induced by the stress. When *F*_*C*−*xz*_ is continuously increasing, the continuing EMT minimizes *y*_*p*_ and leads to a bend in the gastric epithelium. Let us suppose that the heights of the epithelial cells are elevated until the maximum heights are parallel to the height of *C*_*e*_ (*C*_*e*_ refers to the epithelial cell on the MCE site, it does not fixate on any cell) and that mucosal defects due to EMT are repaired by continuous regeneration. Therefore, *F*_*int*_ is zero on the x-axis. Again, supposing that the number of epithelial cells stays essentially the same, some cells are compressed, and the elastic force of these cells counterbalances *F*_*C*−*xz*_. As a result, *F*_*C*−*xz*_ is always in the Euler force vicinity, and EMT can prevent gastric epithelium breakage from continuous regeneration. Therefore, we can write

(32) $$\begin{array}{r} \text{CR}\Rightarrow F_{C-xz}↑\Rightarrow EMT↑\Rightarrow y_{p}↓\Rightarrow F_{C-xz}↓ \end{array}$$

where *CR* is continuous regeneration. However, Equation (32) contains logical paradoxes. The increasing *F*_*C*−*xz*_ and *F*_*C*−*xz*_ always keep themselves in the Euler force vicinity. Now, we need to solve the equation from a different angle: the viscoelastic behavior of macromolecular materials. According to some studies (Hoffman et al., 2006; Fletcher and Mullins, 2010; Guo et al., 2014) on cell mechanics, let us think of these cells as viscoelastic materials. When these cells are compressed, the elastic forces of the cytoskeletons try to return these cells to their original shape. The elastic forces thus must force the cellular liquid back to the original location. However, the recovery time for this process will occur more slowly than deformation because of the liquid's viscosity. The forces that are produced by continuous regeneration are much larger than the elastic force exerted by the cytoskeletons. A combination of large, stiff proteins; protein complexes; smaller, moveable proteins; siRNA (Hannon and Rossi, 2004);microRNA (Ambros, 2004) and other molecules in the cytoplasm is responsible for the process. Suppose that *C*_*e*_ moves distance *S*_*x*_ along the x-axis after the crypt opening demonstrates an approximately stretched line shape and that the first cell division can trigger EMT. According to Hooke's law, we have

(33) $$\begin{array}{r} F_{C-xz}=-KS_{1} \end{array}$$

where *S*_1_ is the displacement distance of *C*_*e*_ along the x-axis after the first cell's division, and *K* is a constant. When our system wants to recover its original shape, there is energy loss in the system because the friction between macromolecules and water produces heat loss. It is impossible for *C*_*e*_ to return to its original position based on energy conservation. Therefore, we have

(34) $$\begin{array}{r} {\;W}_{1}=F_{C-xz}\cdot S_{1}=-\left(F_{v}+Ks_{1}\right)\cdot S_{01} \end{array}$$

where *W*_1_ is the work done along the x-axis after the first cell's division,*F*_*v*_ is the viscous force along the x-axis after the first cell division, and *S*_01_ is the actual moved distance of *C*_*e*_ along the return path. Equations (31) and (32) lead us to

(35) $$\begin{array}{r} {\;S}_{1}>S_{2} \end{array}$$

Using Equation (33), we get

(36) $$\begin{array}{r} S_{x}=\sum_{1}^{n}\left(S_{n}-S_{0n}\right) \end{array}$$

where *n* is the number of times a cell divides after the crypt opening has an approximately stretched line shape. In addition, recent studies (Stolberg and McCloskey, 2009; Mou et al., 2017) have suggested that stem cells' differential behaviors are stimulated by stress. Thus, let us add stress to our model. Therefore, the internal stress is caused by stem cell differentiation within *C*_*e*_'scolumn, which can induce other stem cell differentiation in neighboring columns. The continuous regenerations can trigger EMT at these columns. In the earlier section, it was reasonable to ignore the influences of junctions between *C*_*R*_ and other cells on the MCE. Here, it is important to note that our model is in 3D space. For many cross-sections of the gastric pit, the extent of deformations reduces with their distance from MCE along the y-axis based on Saint-Venant's principle (Hibbeler, 2013). Therefore, we can write

(37) $$\begin{array}{r} |\begin{array}{c} S_{x}=\sum_{1}^{n}\left(S_{n}-S_{0n}\right)\\ S_{x1}=\sum_{1}^{n}\left(S_{n+1}-S_{0\left(n+1\right)}\right)\\ \vdots \\ \vdots \\ S_{xm}=\sum_{1}^{n}\left(S_{m}-S_{0m}\right) \end{array}\;|\;\&S_{x}>S_{x1}\cdots S_{xm} \end{array}$$

where *m* is the number of cross-sections of the gastric pit, and *S*_*xm*_ is the displacement of the stem cell at the isthmus and neck of the gland along the x-axis.

As mentioned above, the value of *F*_*C*−*xz*_ tends to be infinite in our model. Stem cell differentiation is induced by the stress from *F*_*C*−*xz*_ along the x-axis and MCE's drawing force with the rise in MCE. Because the replication zone of the gastric stem cells is at the bottom of a pit, these stem cells can add cells only near the replication zone of the xz-plane because of continuous regeneration. Therefore, we can rewrite Equation (37) as

(38) $$\begin{array}{r} |\begin{array}{c} S_{x}=\sum_{1}^{n}\left(S_{n}-S_{0n}+A_{1}\right)\\ S_{x1}=\sum_{1}^{n}\left(S_{n+1}-S_{0\left(n+1\right)}+A_{2}\right)\\ \vdots \\ \vdots \\ S_{xm}=\sum_{1}^{n}\left(S_{m}-S_{0m}+A_{L}\right) \end{array}\;|\;\&\left(S_{x\left(m-1\right)}>S_{xm}\right)\&\left(D_{L-1}<D_{L}\right) \end{array}$$

Here, *A*_*L*_ is the length of these added cells along the x-axis in different xz-planes. The displacement of *C*_*e*_ in the y-direction is less than or equal to the diameter of a cell. Thus, the geometric shape of our model's gastric pit is very similar to the shape of the lateral sulci.

Based on the simulation and analysis of the motion in the epithelium of the fundic gland, multiple white flat lesions are the result of interactions among four types of mechanisms: continuous regeneration, EMT, the viscoelastic properties of the cells and stem cells induced by stress. First, continuous regeneration provides a tensile force to overstretch crypts at the opening of the fundic gland. Second, stress-induced EMT can prevent gastric epithelium breakage along the y-axis. Third, the viscoelastic properties of cells can “remember” the shape of the cells in the xz-plane. Finally, stem cells induced by stress provide enough epithelial cells to relax the overstretched crypt opening. This complex process is repeated until the initial continuous regeneration stops to produce new epithelial cells. The mechanism through which multiple white flat lesions form can also explain why the crypt opening is approximately linear or a reticular groove on the gastric antrum mucosa.

## Discussion

Can the evolution between the fundic gland and the pyloric gland trigger EMT?

Many physiological cell functions depend on random macromolecular collisions, such as signaling pathways (Strehl and Rohlf, 2016), replication, and transcription (Krebs, 2013). Although we can explain the mechanism of EMT with the help of pathology and some molecular probes (Kalluri and Weinberg, 2009), some unanswered questions still exist in EMT research. For example, can the evolution between the fundic gland and the pyloric gland trigger EMT? If we want to answer this question from our model, we must know a few of the features inside our cells. Recent studies provide evidence (Ellis and Minton, 2003; Margaret et al., 2005) that our cells have a high concentration of molecules, leading to a crowded environment. The characteristics of our cells suggest at least three effects. (1) Proteins are folded into new states in overcrowded conditions (Dhar et al., 2010). When the new state of the protein cause collision with one another, it usually means that a new chemical reaction occurs in the cell. (2) Protein folding is stabilized in overcrowded conditions (Dhar et al., 2010; McGuffee and Elcock, 2010). (3) According to “random walk” (Xiong, 2015), collision probability is increased between molecular motors and microtubules, which has a vast impact on transportation. In addition, intermolecular forces can be classified into three types of force according to their different physical or chemical origins (Israelachvili, 2011). Owing to the crowded environment in the cell and the entropy rule (Phillips et al., 2009), the intermolecular force has a purely entropic origin, such as pressure (stress) or osmotic force.

Let us return to our model. As previously mentioned, when F_C−xz_ is increased, the cell components above the neutral surface are compressed by the stress, and the cell components below it are stretched by the stress in the opposite direction (Figure 7). The polarized nature of these epithelial cells means that the majority of the cytoplasm is located above the neutral surface. Thus, the density of molecules increases locally above the neutral surface. Regarding diffusion, there is a concentration gradient from the region above the neutral surface to the region below the neutral surface. The viscosity can stop the diffusion of macromolecules. However, our cells use molecular motors to overcome this issue. For example, cytoplasmic dynein and kinesin provide controlled directional movement of macromolecules by means of ATP's chemical energy (Schliwa and Woehlke, 2003) in the cytoplasm. ATP is a small molecule, therefore, Its diffusion is not restricted to the cell's viscosity. Let us suppose that ATP is distributed evenly after deformation and ignore the location of the mitochondria. Molecular motors can carry macromolecules along microtubules. Therefore, the contradiction is that if we donot change the direction of the microtubule, we do not keep the cells in mechanical equilibrium (Phillips et al., 2009) based on the entropy rule. Because of spatial asymmetry and because dynein swivels (Rao and Baas, 2017), the osmotic pressure can force cytoplasmic dynein to rotate in a new direction. As a result, most short mobile microtubules point to the cytoplasm below the neutral surface. When these microtubules are transported to the area below the surface by dynein or they elongate into the area via an assembly of tubulins, the dynamic state of plus-end-out microtubules is broken in the lateral direction because the osmotic pressure forces tubulin' s random motion to move along the longitudinal axis according to a collision probability model (Xiong, 2015). In previous studies, microtubule dynamics have a vast impact on the concentration of E-cadherin at cell junctions (Stehbens et al., 2006; Kitase and Shuler, 2013). Our model suggests that a dynamic change in plus-end-out microtubule spatial position induces a change in the sum adhesive power of E-cadherin. In short, the spatial structure's asymmetry leads to EMT. The spatial structure's asymmetry is rooted in the stress from *F*_*C*−*xz*_. Certainly, the details of the relationship between E-cadherin and microtubules are not clearly known, and further studies are needed.

In conclusion, when the external force of cell division acts on our model, kinetic energy can be converted back to potential energy by means of the asymmetrical structure. Potential energy is released with the help of elasticity. Viscosity can be overcome by the interaction of macromolecules and micromolecules. In short, our model suggests that the evolution of the fundic gland and the pyloric gland triggers EMT.

## Author contributions

FX conceived the idea. FX designed and analyzed the model. FX wrote the main manuscript text. XL prepared Figures 1, 4, 5. FX prepared Figures 2, 3, 6, 7. FX prepared all supplementary materials. All authors reviewed the manuscript.

### Conflict of interest statement

The authors declare that the research was conducted in the absence of any commercial or financial relationships that could be construed as a potential conflict of interest.
