# Supplementary material for: A Theoretical Model: Elastic Analysis of the Evolution of the Crypt Opening Between the Fundic Gland and the Pyloric Gland
Source: Front Physiol. 2018 Oct 2;9:1388. doi: 10.3389/fphys.2018.01388 (PMC6190854; doi:10.3389/fphys.2018.01388)
Supplement: Supplementary file 1 [file Presentation_1.ZIP › extended mathematical analysis procedure.docx]

Supplemental Information

Fei Xiong, Xiao Gang Liu

**Extended mathematical analysis procedure**

Suppose that $I$ is the thickness of gastric mucosa, such that $q$ is a given length of gastric mucosa (it is enough to contain the largest length of an colix gastric gland at 2D plane) and$\theta_{k}$ ($k$=1,2,3…n)is the angles between cylinders and $I$. Obviously, the greater the surface area of the cylinders, the more parietal cells and mucus secreting cells in gastric gland. Thus it is easy to understand that more parietal cells and mucus secreting cells mean more gastric acid, so we obtain equation(s1):

$C_{\mathrm{cylinder}}=\sum_{k=1}^{n} S_{k}\frac{q}{{\cos\theta}_{k}}$ (s1)

where $C_{\mathrm{cylinder}}$ is the sum of the surface area of the cylinders except the area of the bottom and the area of the top $S_{k}$ ($k$=1,2,3…n) is the circumference of the cross sectional area of the cylinder,$q$ is the available maximum width of fundic gland in the 2D space(Figure 3). In order to simplify the derivation process of our model and from the point view of superposition principle, let’s suppose these cylinders have the same size of cross section. In addition, owing to the value of$r$is far less than$L$, $r$should also be approximately considered as a constant value. So, In the 2D space(Figure 3), the number of these cylinders of a gastric gland is determined by two factors: the angles between cylinders and mucosal surface-$\theta_{k}$ ($k$=1,2,3…n)and the thickness of gastric mucosa-$L$($L$>>*q*), so we have

$L=\sum_{k=1}^{n} (q\cdot\mathrm{tg}\theta_{k}+{{2r)}}$ (s2)

where $r$is these cylinders’ semi-diameters. Owing to the magnitude of $L$greatly exceeds $r$, $r$ could be approximately considered as both a segment of $L$(a fixed value) and the semi-diameter of cross section of these cylinders.

Now, following the least action principle, we need to find the maximum value of $C_{\mathrm{cylinder}}$ base on some properties of trigonometric function. Take careful note of the form of equation(s1): if each value of$S_{k}$ ($k$=1,2,3…n)is fixed as mentioned above, it is obvious that$C_{\mathrm{cylinder}}$isdetermined by$n$and $\frac{q}{{\cos\theta}_{k}}$. In fact, the essence of principle of least action is extremal problem. We can try to use a mathematical tool-“the first derivative theorem for local extreme values”to find the maximum value of $C_{\mathrm{cylinder}}$.Let’s suppose the value of $\theta_{k}$ ($k$=1,2,3…n)is constant, we have

$C_{\mathrm{cylinder}}=2\pi r\cdot\frac{q}{\cos\theta}\cdot\frac{L}{q\cdot\mathrm{tg}\theta+2r}$ (s3)

If we calculate the derivative of $\frac{q}{{\cos\theta}_{k}}$,we see that

$\left( \frac{q}{\cos\theta} \right)^{'}=q\cdot\frac{\sin\theta}{\cos^{2}\theta}\Longrightarrow\frac{\sin0^{。}}{\cos^{2}0^{。}}=0$ (s4)

It indicates that due to “the first derivative theorem for local extreme values”,$C_{\mathrm{cylinder}}$may have a local maximum value when$\theta$is equal to 0°. According to equation (s3), we have

$C_{\mathrm{cylinder}}=2\pi r\cdot\frac{q}{\cos0^{。}}\cdot\frac{L}{q\cdot\mathrm{tg}0^{。}+2r}=qL\pi$ (s5)

By virtue of these images of gastric body crypt-opening were taken by NBI-ME (Figure 1A), for”0<θ<2/π”$0<\theta<\frac{\pi}{2}$, there exists a mathematical relationship- “q>r” such that

$\frac{\mathrm{qL}}{q\cdot\sin\theta+2cos\theta\cdot r}<\frac{\mathrm{qL}}{r\cdot\sin\theta+2cos\theta\cdot r}<\frac{\mathrm{qL}}{r\cdot\sin\theta+cos\theta\cdot r}$ (s6)

As well known,$\sin\theta+\cos\theta>1$, so we have

$\frac{\mathrm{qL}}{q\cdot\sin\theta+2cos\theta\cdot r}<\frac{\mathrm{qL}}{r}$ (s7)

Then,$qL\pi$ is the maximum value of $C_{\mathrm{cylinder}}$ $C_{\mathrm{cylinder}}$when each angle (θ)has the same value and$\frac{q}{{\cos\theta}_{k}}$ is the height of these cylinders.

Step 2. Let’s suppose that each angle$\theta_{k}$ ($k$=1,2,3…n)has the different values and$\frac{q}{{\cos\theta}_{k}}$is the height of these cylinders. According to equation (s5), $qL$is a rectangle plane’s area, We called it as “QL”.Let’s imagine that these cylinders are different small rectangles (their areas are far less than “QL”, the idea of approximation theory) in the plane(Figure 3). Let’s imagine that each small rectangle is a straight line. It is obvious that$q$, $L$ and $\frac{q}{{\cos\theta}_{k}}$make a triangle(Figure 3), according to the definition of area, we have

$2\sum_{k=1}^{n} q\cdot\mathrm{tg}\theta_{k}+\sum_{k=1}^{n} 2r\cdot\frac{q}{\cos\theta_{k}}=QL=ql$ (s8)

Therefore, it is reasonable to obtain a basic inequality

$\sum_{k=1}^{n} 2r\cdot\frac{q}{\cos\theta_{k}}<ql$ (s9)

Now we tried to multiply equation (s9) by”π”,so we have

$\sum_{k=1}^{n} 2r\pi\cdot\frac{q}{\cos\theta_{k}}<q\pi L$ (s10)

Both side of formula represents these cylinders’ volume under different conditions.

Similarly, the same conclusion is obtained when the available width of fundic gland is less than$q$in the two dimensional space. Step 1 and Step 2 complete the proof of finding the maximum value of $C_{\mathrm{cylinder}}$

Now we describe gastric pit from a viewpoint of approximation. Let’s suppose that gastric pit is a straight tube in the fundus. As well known, gastric pit is a transmitting pipeline and don’t produce gastric acid. We slice gastric pit into many cylinders, and the length of each cylinder approach zero. According to the derivation’s definition, instantaneous velocity is the derivative of position with respect to time. In our model, each cylinder represents instantaneous velocity of the flow of gastric acid. As previously mentioned, the larger the cross section of these cylinders, the higher gastric gland’s transport efficiency. Hence, According to many atlas of magnifying endoscope, let’s suppose that the diameter (D) of crypt-opening of fundic gland is fixed, so it has equation

$D_{\mathrm{opening}}=2r\cdot\cos P\Rightarrow{{(D}_{\mathrm{opening}})}^{`}=-2r\cdot\sin P$ (s11)

where P is the angle between the diameter of crypt-openings and the diameter of gastric pits’ cross section in a 2D plain. According to“the first derivative theorem for local extreme values”, we have

${{(D}_{\mathrm{opening}})}^{`}=-2r\cdot\sin0^{^{\circ}}=0$ (s12)

The equation shows that gastric pit is perpendicular to gastric mucosa on the basis of principle of least action.If $V$ is average efflux velocity of the flow of gastric acid, we obtain

$n=\frac{I-\mathrm{Vt}}{2r}$ (s13)

where t is the time for pouring the flow of gastric acid through gastric pit and “n” is the number of gastric gland’s cylinders (these cylinders are parallel to mucosa surface, they are not gastric pit’s cylinders). As previously mentioned, we need to produce enough gastric acid as soon as possible on the basis of principle of least action, so the value of n approaches maximum. Now let’s suppose t is the only variable in equation(s13), it is obvious that the value of t close to minimum. In addition, the length of gastric pit is determined by t, so we have

$h=\pi r^{2}\cdot v\cdot t$ (s14)

where $h$ is the length of gastric pit. If t is a minimum, it is easy to understand that the length of gastric pit must be short by means of equation (s13). In other word, according to the least action principle and recursion principle, the amount of gastric acid is determine by C_cylinder ,_ C_cylinder_ is determined by n. n is determined by Land h. If Lis fixed, we concluded that normal fundus of stomach has short gastric pit because we need to produce enough gastric acid to filter microbe in a short time.
