# Supplementary material for: A Theoretical Model: Elastic Analysis of the Evolution of the Crypt Opening Between the Fundic Gland and the Pyloric Gland
Source: Front Physiol. 2018 Oct 2;9:1388. doi: 10.3389/fphys.2018.01388 (PMC6190854; doi:10.3389/fphys.2018.01388)

Supplemental Information

Fei Xiong, Xiao Gang Liu

Figure S1 the surface(red) shows an imaginary spatial relationship of Eq(1)’s three variables in three dimensional space.It is obvious that $\frac{\partial E}{\partial t}\cdot\frac{\mathrm{dG}_{\mathrm{ideal}}}{\mathrm{dE}}$ is an extension of $\frac{\mathrm{dG}_{\mathrm{ideal}}}{\mathrm{dt}}$ . as t close to 0, we can easily show $\frac{dG_{\mathrm{real}}}{\mathrm{dt}}$is approximately equal to $\frac{\mathrm{dG}_{\mathrm{ideal}}}{\mathrm{dt}}$because $\frac{\mathrm{dG}_{\mathrm{ideal}}}{\mathrm{dt}}$ is considered starting point. In other word,it is approximately confined to ECM-T plane(a small picture on the top right corner of this picture). In addition,the surface is not the shape in real condition. It may be of any shape.Our goal is irrelevant to its specificity.The shape we choose merely enough to enable us to better understand our model. The meaning of equation(1) of our model is explained as follow:at first, let us suppose that each state of ECM and each state of $G_{\mathrm{ideal}}$ both have an unique state-which is shown by an unique constant.In order to analyze conveniently, we assume that there is one to one between time and these constants (in addition, the conclusion based on it is not limited by the specificity, it can also adapt to any condition, only for analyze conveniently).At second, we need to know the system’s instantaneous rate:$\frac{\mathrm{dG}_{\mathrm{real}}}{\mathrm{dt}}$ . Because of the epithelial-mesenchymal transition andthe continuous regeneration via differentiation of stem cellsplay a keyrolein chronic inflammation, $\frac{\mathrm{dG}_{\mathrm{ideal}}}{\mathrm{dt}}$can be set as the initiating factor for our model. As the multivariable system $G_{\mathrm{real}}$ evolves over time, we should consider that how E affects $\frac{\mathrm{dG}_{\mathrm{ideal}}}{\mathrm{dt}}$. Therefore we need $\frac{\partial E}{\partial t}\cdot\frac{\mathrm{dG}_{\mathrm{ideal}}}{\mathrm{dE}}$ to reduce bias. It is obvious that $\frac{\partial E}{\partial t}\cdot\frac{\mathrm{dG}_{\mathrm{ideal}}}{\mathrm{dE}}$ is an extension of $\frac{\mathrm{dG}_{\mathrm{ideal}}}{\mathrm{dt}}$ in the three dimensional space from the perspective of the geometry


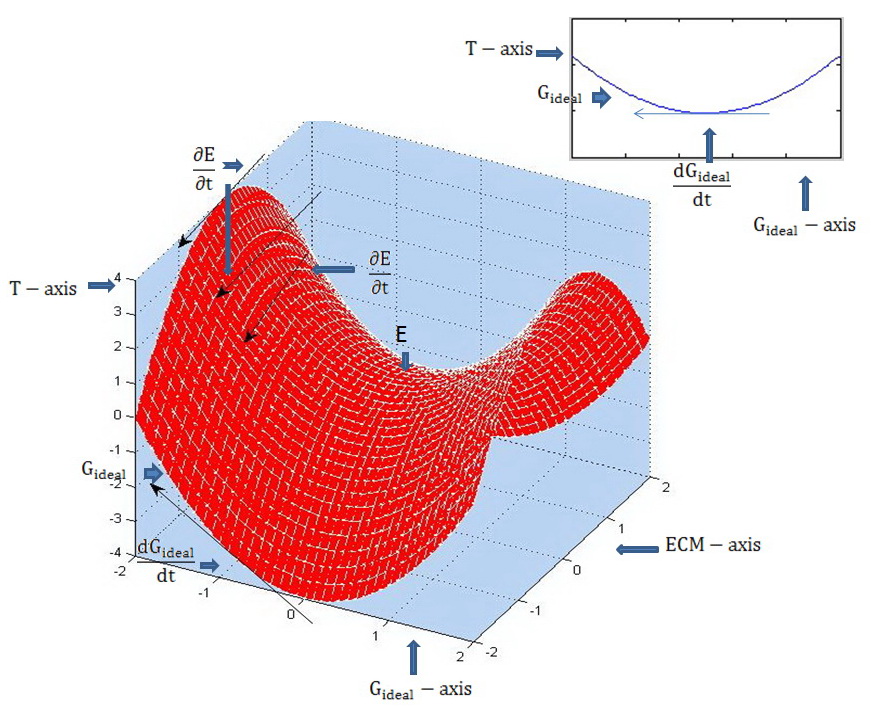

Supplement: Supplementary file 1 [file Presentation_1.ZIP › figure S1.docx]
